# Synergistic potential of probiotics and bacteriophages in combating multidrug-resistant microbial infections: A novel therapeutic strategy for the post-antibiotic era

**DOI:** 10.1016/j.nmni.2026.101804

**Published:** 2026-06-26

**Authors:** Sadaf Ataei-Alamdari, Fatemeh Alimardani, Hamed Afkhami, Mojtaba Kashfi, Mohammad Hasan Yousefi

**Affiliations:** a Master of Medical Bacteriology, Department of Microbiology, Faculty of Medicine, Arak University of Medical Sciences, Arak, Iran; b Master of Medical Bacteriology, Department of Microbiology, Razi Vaccine and Serum Research Institute, Agricultural Research, Education and Extension Organization (AREEO), Karaj, Iran; c Cellular and Molecular Research Center, Qom University of Medical Sciences, Qom, Iran; d Fellowship in Clinical Laboratory Sciences, Qom University of Medical Sciences, Qom, Iran; e Student Research Committee, Qom University of Medical Sciences, Qom, Iran; f Department of Tissue Engineering and Applied Cell Sciences, School of Medicine, Qom University of Medical Sciences, Qom, Iran

**Keywords:** Probiotics, Bacteriophages, Antibiotic resistance, Phage therapy, MDR pathogens, Microbiome modulation, Combination therapy

## Abstract

In recent years, the rapid emergence of multidrug-resistant (MDR) pathogens has posed a global health crisis, necessitating the exploration of alternative therapeutic strategies beyond conventional antibiotics. Among emerging solutions, the combination of probiotics and bacteriophages has gained significant attention due to their complementary mechanisms in targeting pathogenic bacteria while preserving host microbiota. This review comprehensively evaluates the molecular interactions, synergistic effects, safety profiles, and clinical applicability of probiotic-phage combinations in managing MDR infections. We also discuss challenges related to formulation, delivery, regulatory considerations, and future directions for translating this approach into clinical practice. The integration of probiotics and phage therapy represents a promising avenue to address antibiotic resistance, offering a personalized, targeted, and microbiome-friendly antimicrobial strategy.

## Highlights

• Probiotics and bacteriophages act synergistically to combat multidrug-resistant pathogens.
• The combination preserves microbiome integrity while enhancing pathogen clearance.
• Preclinical models show superior efficacy of combined therapy over monotherapies.
• Formulation strategies and regulatory pathways for clinical translation are reviewed.

## 1 Introduction

### 1.1 Global burden of antimicrobial resistance (AMR)

The magnitude of antimicrobial resistance as a global health threat cannot be overstated, with recent comprehensive assessments revealing alarming trends in mortality and morbidity worldwide. In 2021, bacterial AMR was associated with 4.71 million deaths globally, including 1.14 million deaths directly attributable to resistance mechanisms [1]. Projections suggest that by 2050, AMR could cause 10 million deaths annually, with significant economic burdens, particularly in low- and middle-income countries. The World Bank estimates that by 2030, AMR could cost the global economy up to US$100 trillion [2]. These figures represent not merely statistical abstractions but a profound human tragedy that continues to unfold across all geographic regions and demographic groups. The economic and social implications of this crisis extend far beyond immediate healthcare costs, encompassing reduced productivity, prolonged hospitalizations, and the erosion of medical advances that depend on effective antimicrobial therapy. Projections for the coming decades paint an even more concerning picture, with forecasting models indicating that AMR-related deaths could increase by 69.6% from 2022 to 2050 [1]. This dramatic escalation reflects both the continued emergence of resistant strains and the aging global population, which is particularly vulnerable to resistant infections. The burden is not uniformly distributed across age groups, with concerning trends showing increased AMR mortality among individuals older than 70 years, while some improvement has been observed in children under 5 years due to enhanced infection prevention measures. These demographic patterns underscore the complex interplay between population dynamics, healthcare infrastructure, and antimicrobial stewardship in shaping resistance outcomes [1].

### 1.2 Limitations of conventional antibiotics

The pharmaceutical landscape for antibiotic development faces unprecedented challenges that fundamentally undermine the traditional model of antimicrobial innovation. Economic factors play a decisive role in shaping industry priorities, with antibiotic development suffering from poor performance in economic decision models compared to drugs for chronic diseases [3]. The trend toward more severe and widespread market restrictions for antibiotic use, implemented ostensibly to control resistance but often enacted through drug budget controls, significantly reduces the potential earnings of new antibiotics [3]. This economic reality creates a paradoxical situation where the urgent need for new antimicrobials conflicts with the financial disincentives that discourage pharmaceutical investment in this area. Regulatory and developmental challenges compound these economic barriers, with increasingly stringent approval procedures elevating both development costs and associated risks [3]. On the other hand The current antibiotic pipeline is failing to meet clinical needs due to rapid emergence of resistance and reduced pharmaceutical investment. Most antibiotics introduced in the past two decades are variations of existing classes, and novel drug classes are scarce [4]. The misuse of antibiotics in human medicine and agriculture has further accelerated resistance, leaving multidrug-resistant (MDR) pathogens like *Acinetobacter baumannii*, *Klebsiella pneumoniae*, and *Pseudomonas aeruginosa* as major threats [5]. The clinical limitations of conventional antibiotics extend beyond resistance mechanisms to encompass issues of spectrum, toxicity, and ecological impact. Broad-spectrum antibiotics, while offering therapeutic convenience, indiscriminately target beneficial bacterial populations alongside pathogenic organisms, potentially disrupting critical microbial ecosystems that contribute to host health and resistance to colonization by opportunistic pathogens. For instance, broad-spectrum agents such as fluoroquinolones and carbapenems can cause rapid depletion of obligate anaerobes (e.g., *Bacteroides*, *Faecalibacterium*), reduce short-chain fatty acid production, and create vacant ecological niches that permit opportunistic pathogens like *Clostridioides difficile* or vancomycin-resistant *Enterococci* to proliferate. Longitudinal studies show that even short antibiotic courses can alter microbiota composition for months, impairing colonization resistance and increasing susceptibility to secondary infections [6,7]. Furthermore, the inevitable development of resistance to any antimicrobial agent means that even the most innovative conventional antibiotics face an inherently limited therapeutic lifespan, necessitating continuous investment in drug discovery that current market dynamics cannot sustainably support [8].

### 1.3 Emergence of alternative therapies

The recognition of conventional antibiotic limitations has catalyzed unprecedented interest in alternative therapeutic modalities that can circumvent traditional resistance mechanisms while offering sustainable treatment options for the post-antibiotic era. These innovative approaches represent a fundamental shift from the paradigm of broad-spectrum bacterial killing toward more targeted, mechanistically diverse strategies that can complement or replace conventional antimicrobials. The development of alternative therapies encompasses multiple distinct approaches, each offering unique advantages and addressing specific limitations of traditional antibiotic therapy [8]. Phage therapy has emerged as one of the most promising alternatives, utilizing bacteriophages—viruses that specifically infect and kill bacteria—to treat bacterial infections with remarkable precision [8,9]. Unlike antibiotics, which often affect broad spectra of bacteria including beneficial microbiota, phages demonstrate exquisite specificity for their bacterial targets, minimizing collateral damage to the host's microbial community. This specificity, combined with phages' ability to evolve alongside their bacterial targets, offers a dynamic therapeutic approach that can potentially overcome the static nature of conventional antimicrobials [8]. Probiotic interventions represent another compelling alternative strategy, harnessing the power of beneficial microorganisms to compete with pathogenic bacteria for resources and colonization sites within the host. By promoting balanced microbial communities and enhancing natural defense mechanisms, probiotics offer a preventive and therapeutic approach that strengthens host resistance rather than relying solely on pathogen elimination [8]. Immunomodulatory therapies constitute a third major category of alternatives, focusing on enhancing the host's immune response rather than directly targeting microorganisms. These approaches, including cytokines, monoclonal antibodies, and vaccines, aim to reduce dependence on antimicrobial agents by bolstering natural immune surveillance and response mechanisms [8].

### 1.4 Historical context of phage therapy and probiotic use

#### 1.4.1 Phage therapy development

The historical trajectory of phage therapy reveals a fascinating narrative of early promise, subsequent decline, and contemporary resurgence that mirrors broader trends in antimicrobial therapeutics. The discovery of bacteriophages and their therapeutic potential can be traced to the pioneering work of Frederick Twort in 1915, who first suggested that viruses were responsible for the bacterial-killing "factor" that had been previously observed [10]. Felix d'Herelle subsequently expanded upon this work, proposing phages as therapeutic agents for human infections and conducting the first documented therapeutic application in 1919. The early applications of phage therapy demonstrated remarkable clinical success, exemplified by d'Herelle's treatment of a 12-year-old boy with severe dysentery using a phage cocktail, resulting in complete symptom resolution within days [10]. Despite such promising results, phage therapy faced significant scientific and practical challenges that ultimately led to its decline in Western medicine during the antibiotic era. Controversies surrounding the biological nature of phages, combined with the convenience and broad-spectrum activity of newly discovered antibiotics, contributed to the marginalization of phage research in most countries outside the Soviet Union [10]. The Soviet Union and, to some extent, the German army during World War II continued to develop and employ phage therapy even as Western medicine abandoned this approach. This divergent path created a unique situation where Eastern European countries maintained extensive experience with phage applications while Western pharmaceutical development focused exclusively on chemical antimicrobials [10]. The contemporary resurgence of interest in phage therapy reflects both the urgent need for alternatives to failing antibiotics and the accumulated knowledge from decades of continued research and clinical application in countries that never abandoned this therapeutic modality [10].

#### 1.4.2 Probiotic evolution

The evolution of probiotics as a therapeutic tool has undergone significant advancements since 2018, driven by innovations in microbiology, synthetic biology, and microbiome research. Probiotics, defined as live microorganisms that confer health benefits when administered in adequate amounts, have expanded from traditional gut health applications to promising interventions against multidrug-resistant (MDR) microbial infections [11]. Recent research has elucidated the multifaceted mechanisms by which probiotics combat MDR pathogens. Probiotics exert antimicrobial effects through competitive exclusion, production of bacteriocins, and modulation of the host immune system. For instance, *Lactobacillus rhamnosus* GG has been shown to reduce *Staphylococcus aureus* carriage in veterans, demonstrating its potential to mitigate MDR infections through mucus-binding pili and competitive niche occupation [12]. Similarly, *Lactobacillus paracasei* CNCM I-3689 promotes gut microbiota resilience and reduces vancomycin-resistant *Enterococcus* (*VRE*) persistence following antibiotic challenge [13]. Probiotics may also interfere with biofilm formation, an important contributor to persistence and antimicrobial resistance in MDR infections. *Lactobacillus rhamnosus* formulations have shown efficacy against methicillin-resistant *Staphylococcus aureus* (MRSA) and *VRE* by inhibiting biofilm formation and reducing pathogen toxicity on host cell [14,15]. Synthetic biology has transformed probiotic development by enabling the engineering of strains with targeted antimicrobial properties. Engineered *Lactobacillus reuteri* expressing antimicrobial peptides has demonstrated efficacy in reducing *Staphylococcus aureus* colonization in preclinical models [16]. Similarly, CRISPR-based gene editing has been utilized to enhance probiotic strains' ability to produce phage-derived enzymes, increasing their antimicrobial potency against MDR pathogens [17,18]. These advancements allow probiotics to deliver precise therapeutic payloads, such as enzymes that degrade bacterial cell walls or inhibit efflux pumps, which are key resistance mechanisms in pathogens like *Pseudomonas aeruginosa* [19].

### 1.5 Rationale for combining probiotics and phages

Probiotics and phages operate through distinct yet complementary mechanisms, making their combination a powerful tool against MDR pathogens. Probiotics, such as *Lactobacillus* and *Bifidobacterium* species, enhance host defenses by strengthening mucosal barriers, producing antimicrobial compounds like bacteriocins, and competitively excluding pathogens from ecological niches [16]. For example, *Lactobacillus rhamnosus* GG has been shown to reduce *Staphylococcus aureus* colonization by inhibiting adhesion and biofilm formation [14]. In contrast, bacteriophages are viruses that specifically infect and lyse target bacteria, offering precision in eliminating MDR pathogens without disrupting the broader microbiome [20]. Phages can penetrate biofilms, a common resistance mechanism in pathogens like *Pseudomonas aeruginosa*, and disrupt bacterial cell integrity [21]. The combination of probiotics and phages enhances therapeutic efficacy by simultaneously reducing pathogen load (via phage lysis) and restoring microbial balance (via probiotic activity). A 2018 study demonstrated that alginate-encapsulated *Lactobacillus* strains, when combined with phages and tobramycin, eradicated MRSA and *P. aeruginosa* in wound infection models by leveraging probiotics' lactic acid production and phages’ targeted lysis [22]. The temporal dynamics of phage and probiotic action create opportunities for synergistic effects that enhance overall therapeutic efficacy. Phage therapy typically provides rapid reduction in pathogenic bacterial loads through direct lysis, creating ecological niches that can be rapidly colonized by beneficial probiotic strains [8]. This sequential or concurrent application prevents the reestablishment of pathogenic populations while promoting the restoration of healthy microbial communities that provide long-term resistance to reinfection. Furthermore, the specificity of phage action ensures that probiotic strains remain unaffected by therapeutic phage administration, allowing for sustained probiotic effects throughout the treatment period [8]. The resistance profiles of combined phage-probiotic therapies offer significant advantages over conventional antimicrobial approaches. While bacteria may develop resistance to specific phages through various mechanisms, the presence of competing probiotic populations can limit the fitness advantages of phage-resistant mutants, potentially slowing the emergence of resistance [8]. Additionally, the use of phage cocktails targeting multiple bacterial proteins, combined with probiotic-mediated competitive pressure, creates multiple selective pressures that make the evolution of broadly resistant phenotypes significantly more challenging than resistance to single antimicrobial agents (Table 1) [8]. The synergistic potential of probiotic-phage combinations extends beyond additive effects, representing a true 1 + 1>2 therapeutic paradigm. Unlike conventional antibiotics that indiscriminately eliminate both pathogens and commensals, phages provide precision lysis while probiotics actively restore ecological balance. Mechanistically, probiotics weaken biofilm matrices and reduce pathogen adhesion, significantly enhancing phage penetration and access to target cells. Concurrently, phage-mediated pathogen clearance reduces competitive pressure, allowing probiotics to colonize more efficiently and secrete higher levels of immunomodulatory metabolites. This bidirectional reinforcement creates a self-sustaining therapeutic loop that outperforms either modality alone, minimizes resistance selection, and preserves microbiome integrity [[23], [24], [25]].

**Table 1:** Comparative features of probiotics and bacteriophages.

Feature	Probiotics	Bacteriophages
Specificity	Moderate	High
Mode of Action	Metabolite secretion, immune modulation	Lysis, enzyme release
Host Range	Broad spectrum	Narrow or specific
Resistance Development	Low	Possible (host adaptation)
Safety Profile	Generally safe	Requires careful monitoring
Application Route	Oral, topical	Oral, IV, topical

#### 1.5.1 Objective and scope of the review

This comprehensive review aims to evaluate the scientific evidence supporting the synergistic potential of probiotics and bacteriophages in combating multidrug-resistant bacterial infections, with particular emphasis on mechanistic understanding, clinical applications, and future therapeutic development. The primary objective encompasses a systematic analysis of existing literature documenting combined phage-probiotic interventions, including both preclinical studies and clinical applications, to establish the current state of knowledge regarding this therapeutic combination. The review will critically assess the biological basis for synergistic effects, examining molecular mechanisms, ecological interactions, and immunological responses that contribute to enhanced therapeutic outcomes when these modalities are used in combination. The scope of this review extends beyond simple efficacy assessments to encompass the practical considerations necessary for clinical translation of combined phage-probiotic therapies. This includes evaluation of formulation challenges, delivery systems, dosing strategies, and safety profiles that must be addressed to enable widespread clinical implementation. The review will also examine regulatory considerations specific to combination therapies that cross traditional pharmaceutical boundaries, incorporating both biological and live microbial therapeutic products within single treatment regimens. Furthermore, this review will address the broader implications of combined phage-probiotic therapy for antimicrobial stewardship and resistance prevention strategies. The analysis will consider how these alternative approaches might be integrated into existing treatment protocols, their potential role in preventing healthcare-associated infections, and their contribution to global efforts to combat antimicrobial resistance. Special attention will be given to identifying research gaps and priority areas for future investigation that could accelerate the clinical development and implementation of these promising therapeutic combinations.

## 2 Mechanisms of action

### 2.1 Probiotics

Key probiotic mechanisms relevant to combating MDR infections include:

#### 2.1.1 Competitive exclusion

Probiotics directly compete with pathogens for essential nutrients (e.g., iron, carbohydrates) and adhesion sites on the mucosal epithelium and mucus layer. By occupying these ecological niches, they prevent pathogen colonization and establishment, a crucial first step in infection pathogenesis. This mechanism is intrinsically resistant to the pathogen's acquired antibiotic resistance profile [26,27].

#### 2.1.2 Production of antimicrobial metabolites

Probiotics synthesize a diverse arsenal of bioactive compounds with direct inhibitory or lethal effects on competing microbes. These include.

#### 2.1.3 Organic acids (e.g., lactic, acetic, propionic)

Lowering environmental pH, disrupting membrane potential, and denaturing pathogen proteins [28].

#### 2.1.4 Bacteriocins

Ribosomally synthesized peptides or proteins (e.g., nisin, plantaricin, reuterin) with often narrow spectra, targeting specific pathogens through mechanisms like pore formation or cell wall synthesis inhibition, frequently bypassing classical resistance mechanisms [29,30].

#### 2.1.5 Hydrogen peroxide (H_2_O_2_)

Generating reactive oxygen species that cause oxidative damage, particularly effective against anaerobes and catalase-negative bacteria [28].

#### 2.1.6 Short-chain fatty acids (SCFAs)

Metabolic byproducts (e.g., butyrate, propionate, acetate) with pleiotropic effects, including direct antimicrobial activity, lowering colonic pH, and enhancing barrier function [31].

#### 2.1.7 Immune modulation and enhancement of mucosal barrier function

Probiotics interact dynamically with host immune cells (e.g., dendritic cells, macrophages) and epithelial cells, leading to.

#### 2.1.8 Strengthened epithelial barrier

Upregulation of mucus production (mucins like MUC2), enhancement of tight junction protein expression (e.g., ZO-1, occludin), and promotion of epithelial cell survival, reducing paracellular permeability and pathogen translocation [32,33].

#### 2.1.9 Regulated immune responses

Modulation of innate and adaptive immunity, including balanced cytokine production (e.g., promoting anti-inflammatory IL-10, regulating pro-inflammatory signals), stimulation of antimicrobial peptide (e.g., defensins) secretion, and enhancement of secretory IgA (sIgA) production, facilitating pathogen neutralization [34,35].

#### 2.1.10 Quorum sensing interference (quorum quenching)

Probiotics can disrupt bacterial cell-to-cell communication systems (Quorum Sensing - QS) essential for coordinating virulence factor expression (e.g., toxin production, biofilm formation) and pathogenicity in a density-dependent manner. Mechanisms include producing quorum sensing inhibitors (QSIs) that mimic or block autoinducer signals, or secreting enzymes (e.g., lactonases, acylases) that degrade these signaling molecules. This "anti-virulence" strategy attenuates pathogenicity without directly killing the bacteria, potentially reducing selective pressure for resistance development.

Concurrently, bacteriophages (phages) – viruses that specifically infect and lyse bacteria – have experienced a significant resurgence as precision antimicrobials. Their ability to co-evolve with bacteria, target specific strains (including MDR clones), penetrate biofilms, and replicate at the site of infection offers unique advantages, particularly where antibiotics fail [36,37]. While both probiotics and phages individually show considerable promise against MDR pathogens, their therapeutic potential may be significantly amplified through synergistic interactions. Probiotics can modulate the host environment and pathogen behavior in ways that enhance phage efficacy (e.g., reducing pathogen load via competitive exclusion or metabolites, disrupting biofilms via quorum quenching, modulating immune clearance), while phages can selectively eliminate target MDR pathogens that might otherwise outcompete or resist probiotic activity. Conversely, probiotics may help mitigate potential inflammatory responses or dysbiosis associated with phage-mediated lysis [38,39].

### 2.2 Bacteriophages

Bacteriophages, or phages, are viruses that specifically infect bacteria, offering a promising alternative to traditional antibiotics in the fight against multidrug-resistant (MDR) bacterial infections. With the global rise of antibiotic resistance, responsible for approximately 1.27 million deaths in 2019 according to the World Health Organization, phages have re-emerged as a potential therapeutic tool [40].

#### 2.2.1 Specificity and lytic activity against target bacteria

Bacteriophages exhibit remarkable specificity, often infecting only a single bacterial species or specific strains within a species. This precision stems from the interaction between phage receptor-binding proteins (RBPs), such as tail fibers or tail spikes, and specific bacterial surface receptors, including lipopolysaccharides (LPS), outer membrane proteins (OMPs), or teichoic acids in Gram-positive bacteria [41]. For instance, studies have shown that mutations in the T4 phage gp37 protein enable it to attach to *Escherichia coli O157* receptors, while chimeric phages combining tail proteins from different phages can redirect specificity to new hosts like *Salmonella.* This specificity minimizes disruption to the host microbiota, reducing the risk of dysbiosis compared to broad-spectrum antibiotics [41]. Upon attachment, lytic phages inject their genetic material into the host bacterium, utilizing the host's ribosomes to produce viral proteins and genomes. The host cell's resources are rapidly converted into new phage particles, culminating in cell lysis and the release of progeny phages, which can infect additional bacteria [40]. This lytic cycle is particularly effective against MDR bacteria, as demonstrated in clinical cases where phages successfully treated infections caused by *Pseudomonas aeruginosa* and *Acinetobacter baumannii* [40]. However, bacterial resistance mechanisms, such as CRISPR-Cas9 or receptor modification, pose challenges, prompting research into phage engineering to broaden host range and overcome resistance [41].

#### 2.2.2 Biofilm disruption and penetration

Bacterial biofilms, complex communities of bacteria encased in an extracellular polymeric substance (EPS) matrix, are notoriously resistant to antibiotics and immune responses, contributing to chronic infections such as those associated with medical implants or cystic fibrosis. Phages can disrupt biofilms through multiple mechanisms. Some phages naturally produce depolymerases that degrade EPS components, such as polysaccharides, enabling penetration and access to embedded bacteria [42]. For example, the phage DW-EC has been shown to degrade biofilms of *E. coli O157:H7*, reducing biofilm formation by up to 82.4% [43]. Engineered phages enhance this capability by expressing enzymes like DspB, which targets β-1,6-N-acetyl-D-glucosamine, a key EPS component. In a study, the engineered phage T7 DspB reduced *E. coli* biofilm cell counts by approximately 99.997% (4.5 log_10_ CFU per peg), significantly outperforming non-enzymatic phages [44]. Additionally, combining phages with antibiotics or disinfectants, such as chlorine, has shown synergistic effects, further enhancing biofilm disruption by compromising bacterial cell membranes. These properties make phages a potent tool for managing biofilm-associated infections, though challenges like phage resistance and delivery optimization remain [42].

#### 2.2.3 Enzybiotic potential

Phage-encoded endolysins, also known as enzybiotics, are peptidoglycan hydrolases that degrade bacterial cell walls, leading to osmotic lysis and cell death. These enzymes are naturally produced by phages at the end of the lytic cycle to release progeny virions but can be applied exogenously as recombinant proteins to kill bacteria, including MDR strains [45]. Endolysins from Gram-positive phages typically have a modular structure, consisting of enzymatically active domains (EADs) that cleave peptidoglycan bonds and cell wall binding domains (CBDs) that ensure specificity. For example, the endolysin PlyC reduced *Streptococcus* colonization to less than 30% in mouse models, while LysH5 eliminated *Staphylococcus aureus* in milk within 4 h [45]. Endolysins are highly specific, targeting only certain bacterial genera or species, which minimizes disruption to commensal flora and reduces resistance development compared to antibiotics. Studies with endolysins like Pal, PlyG, and ClyS against *S. pneumoniae*, *B. anthracis*, and MRSA, respectively, reported no resistance, highlighting their robustness [45]. Recent engineering efforts have expanded their utility to Gram-negative bacteria by fusing endolysins with cationic peptides that penetrate the outer membrane, broadening their therapeutic potential [45]. Applications extend beyond medicine to food safety (e.g., reducing *Listeria monocytogenes* on lettuce) and agriculture (e.g., protecting crops from *Erwinia carotovora*), underscoring their versatility [45].

#### 2.2.4 Modulation of host immunity via phage-host interaction

Beyond their antibacterial effects, bacteriophages interact with the mammalian immune system, modulating inflammatory and immune responses in ways that may enhance therapeutic outcomes. Phages can reduce pro-inflammatory cytokines such as IL-6, IL-8, TNF-α, and IL-1β while upregulating anti-inflammatory factors like IL-10 and SOCS3, as observed in models of lung infections, urinary tract infections, and skin graft transplants [46] or instance, T4 phage gp12 binds LPS, reducing IL-1α and IL-6 levels, and *P. aeruginosa* phages decrease pro-inflammatory signals via the TLR-MyD88 pathway [46]. Phages also influence immune cell behavior, such as macrophage polarization, and can trigger antiviral responses via TLR3, leading to type 1 interferon production that indirectly affects bacterial infections [47]. However, immune responses vary by phage strain, dosage, and administration route, with high titers (e.g., 10^9 PFU/well) sometimes inducing pro-inflammatory effects. While these immunomodulatory effects suggest potential for treating inflammatory conditions, the lack of clinical trials and undefined pharmacology highlight the need for further research to ensure safety and efficacy [46].

### 2.3 Synergistic interactions

**Fig. 1:**
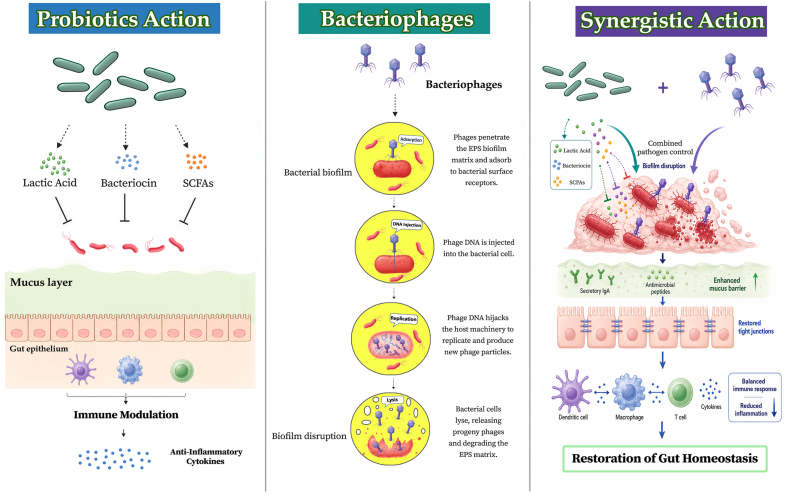
Schematic representation of the distinct and synergistic mechanisms of probiotics, bacteriophages, and their combination in combating multidrug-resistant (MDR) pathogens. **Left panel:** Probiotics exert their effects through increased mucus layer production, secretion of antimicrobial metabolites (e.g., lactic acid, bacteriocins), and modulation of immune responses via anti-inflammatory cytokines, ultimately restoring epithelial barrier integrity. **Middle panel:** Bacteriophages specifically target MDR bacteria through adsorption to surface receptors, injection of genetic material, and enzymatic degradation of biofilms (via depolymerases), culminating in bacterial lysis. **Right panel:** The synergistic combination leverages both modalities: phages perform targeted lysis of pathogenic bacteria within biofilms, while probiotics simultaneously restore the mucosal layer, reduce inflammation, and reinforce the epithelial barrier, creating an environment resistant to reinfection. This dual-action approach offers a microbiome-friendly strategy for overcoming antibiotic resistance.

The integration of probiotics with bacteriophages has emerged as a novel strategy to combat MDR infections (Fig. 1). Probiotics restore microbial balance and enhance host defenses, while bacteriophages selectively target resistant pathogens. A 2018 study by MIT researchers demonstrated that alginate-encapsulated *Lactobacillus* strains, combined with tobramycin, eradicated MRSA and *Pseudomonas aeruginosa* in wound infection models by leveraging probiotics' lactic acid production and phages' targeted bacterial lysis [22]. Similarly, co-administration of *Lactobacillus* species with bacteriophages reduced *Clostridium difficile* colonization in vitro and in vivo, suggesting a model for synergistic MDR infection management [21]. At the molecular level, probiotic-derived metabolites such as lactic acid, SCFAs, and bacteriocins can lower local pH and alter bacterial membrane fluidity, potentially enhancing phage receptor accessibility and adsorption efficiency. Conversely, phage-mediated lysis releases intracellular nutrients and cell wall fragments that may stimulate probiotic proliferation or modulate host pattern-recognition receptors (e.g., TLR2/4, NOD-like receptors). Furthermore, probiotic-induced upregulation of secretory IgA and antimicrobial peptides can synergize with phage clearance mechanisms, creating a multi-layered molecular defense network against MDR pathogens [[48], [49], [50], [51]].

#### 2.3.1 Enhanced efficacy through combined colonization and lysis

The synergy between probiotics and phages has shown superior outcomes compared to monotherapies in preclinical and clinical settings. For instance, co-administration of *Lactobacillus* species with bacteriophages significantly reduced *Clostridium difficile* colonization in both in vitro and in vivo models, with probiotics enhancing gut microbiota resilience and phages directly targeting the pathogen [21]. Together, these findings support the rationale for combining microbiota-modulating probiotics with pathogen-specific bacteriophages, as probiotics may enhance colonization resistance and host–microbiota homeostasis, while phages provide targeted bacterial killing [4]. Also studies have demonstrated that phage–antibiotic synergy, which can be extended to phage–probiotic combinations, significantly reduces bacterial biofilms, particularly in older, more resistant biofilms. For instance, a combination of phages and *Lactobacillus* species has shown enhanced biofilm disruption in *S. aureus* models, where probiotics weaken the biofilm matrix, and phages penetrate to lyse embedded bacteria [52].

#### 2.3.2 Modulation of gut and skin microbiota to prevent dysbiosis

One of the critical advantages of phage–probiotic combinations is their ability to minimize dysbiosis, a common side effect of broad-spectrum antibiotics. Antibiotics often disrupt the gut and skin microbiota, leading to conditions like *Clostridioides difficile* colitis or increased susceptibility to secondary infections. Phages, with their high specificity, target only the pathogenic bacteria, sparing beneficial microbiota. Probiotics, such as *Bifidobacterium* and *Lactobacillus* species, further support microbial balance by promoting the growth of commensal bacteria and enhancing the gastrointestinal and skin barriers. A 2021 study demonstrated that *Lactobacillus rhamnosus* GG supplementation reduced the duration of diarrhea and improved gastrointestinal function in patients with rotavirus-associated infections, highlighting its role in restoring gut homeostasis. Similarly, phage therapy combined with probiotics has shown promise in maintaining skin microbiota integrity during wound infections, reducing the risk of chronic infections [53,54].

## 3 Preclinical evidence of Probiotic–Phage synergy

### 3.1 In vitro studies on biofilm disruption and bacterial clearance

**Fig. 2:**
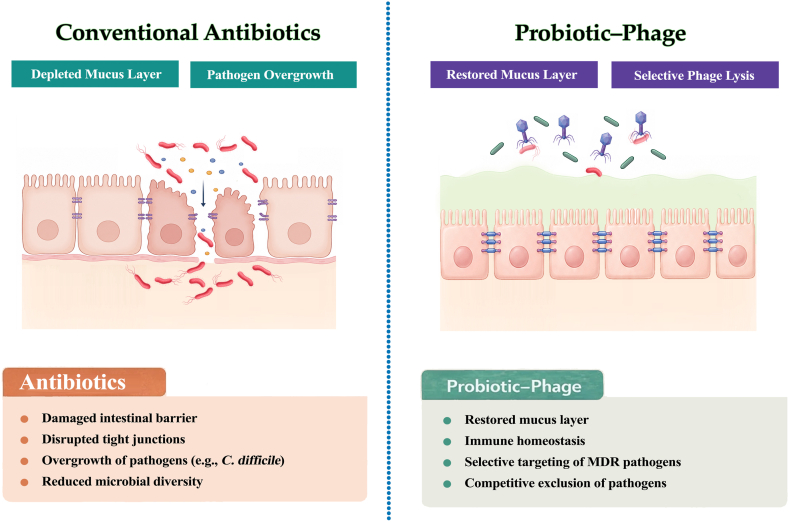
Comparative impact of conventional antibiotics versus combined probiotic–phage therapy on host microbiota and tissue integrity. **Left panel (Conventional Antibiotics):** Broad-spectrum antibiotics disrupt the intestinal barrier, damage tight junctions, deplete the mucus layer, and cause overgrowth of opportunistic pathogens like *Clostridioides difficile*, leading to reduced microbial diversity and dysbiosis. **Right panel (Probiotic–Phage Therapy):** This targeted approach restores the mucus layer, promotes immune homeostasis, and enables selective lysis of MDR pathogens without harming commensal bacteria. The resulting competitive exclusion of pathogens and reinforcement of the epithelial barrier create a sustainable, microbiome-friendly therapeutic outcome. This paradigm shift offers a promising solution to the limitations of traditional antimicrobial strategies in the post-antibiotic era.

Biofilms, structured communities of bacteria encased in extracellular matrices, are a major contributor to antibiotic resistance, particularly in chronic infections. In vitro studies have demonstrated that combining probiotics with phages enhances biofilm disruption and bacterial clearance. For example, a study on *S. aureus* biofilms showed that a combination of *Lactobacillus plantarum* and a staphylococcal phage (Sb-1) significantly reduced biofilm biomass compared to either treatment alone. The probiotic's production of lactic acid and bacteriocins weakened the biofilm matrix, allowing the phage to access and lyse embedded bacteria. Similarly, a 2023 study found that a cocktail of *E. coli* targeting phages combined with *Bifidobacterium bifidum* enhanced bacterial clearance in a gut infection model, with probiotics inhibiting pathogen adhesion and phages reducing bacterial viability [55,56]. Another study investigated the synergistic effects of *a Pseudomonas aeruginosa* phage cocktail (PAM2H) and a probiotic mixture containing *Lactobacillus* and *Bifidobacterium* species in a burn wound model. The combination reduced biofilm formation by 5.5 log units compared to 3.5 log units for phages alone, demonstrating enhanced efficacy. These findings underscore the potential of probiotic–phage combinations to overcome the challenges posed by biofilms, which conventional antibiotics often fail to penetrate (Fig. 2) [52]. Ecologically, the probiotic-phage combination promotes microbiome resilience by restoring niche occupancy and metabolic redundancy. Phages selectively reduce pathogen biomass without depleting commensal taxa, while probiotics rapidly occupy freed ecological niches, preventing secondary opportunistic overgrowth. Network analysis of microbial communities suggests that this dual approach accelerates the recovery of alpha-diversity and stabilizes keystone taxa involved in barrier function and immune homeostasis, offering a sustainable ecological alternative to broad-spectrum antimicrobials [[57], [58], [59]].

#### 3.1.1 Animal models of infection

Animal models have further validated the efficacy of probiotic–phage synergy in various infection types.

#### 3.1.2 Wound infections

A murine model of *S. aureus* wound infections demonstrated that a combination of *Lactobacillus acidophilus* and a staphylococcal phage cocktail reduced bacterial load by 4 log units compared to 2 log units with phages alone. The probiotic enhanced epithelial barrier function, while the phages targeted MDR *S. aureus*, preventing infection progression. Similarly, a 2024 study in a mouse burn wound model showed that a *P. aeruginosa* phage cocktail (PP1131) combined with a probiotic mixture (*Lactobacillus* and *Bifidobacterium* species) achieved a 90% reduction in bacterial burden, compared to 60% with phages alone [53,56].

#### 3.1.3 Urinary tract infections (UTIs)

In a rat model of *E. coli* induced UTIs, a combination of *Lactobacillus rhamnosus* GR1 and a CRISPR-Cas3-enhanced phage cocktail (LBP-EC01) eradicated 80% of the bacterial load, compared to 50% with phages alone. The probiotic inhibited *E. coli* adhesion to bladder epithelial cells, while the phage lysed the bacteria, preventing recurrence [60,61].

#### 3.1.4 Gastrointestinal infections

In a hamster model of *Clostridium difficile* infection, the combined oral administration of *Bifidobacterium longum* as a probiotic and a *C. difficile*-specific phage cocktail may offer a synergistic therapeutic effect. The probiotic could help restore gut microbiota diversity by promoting beneficial microbial populations [62,63], while the phage cocktail would directly target and lyse *C. difficile* cells, reducing bacterial proliferation and toxin-mediated damage [64,65]. This dual therapy strategy has the potential to prevent mortality associated with antibiotic-resistant *C. difficile* infections by simultaneously modulating the microbiota and providing targeted bacteriolytic activity, as suggested by previous studies on probiotics and phage therapy in gastrointestinal infections [62,65]. Also 2025 study demonstrating *Bifidobacterium breve* synergy with other microbes in a murine model, which restored microbiota diversity and reduced inflammation [66]. A 2024 study in a murine gut model investigated the synergy between a mucus-adherent *Escherichia coli* phage (øPNJ-6) and commensal gut microbes. The phage targeted enterotoxigenic *E. coli* (ETEC), reducing its abundance while allowing commensal bacteria like *Klebsiella oxytoca* to proliferate, enhancing gut microbial balance. Pre-treatment with øPNJ-6 led to increased mucus production and improved host cell viability, suggesting a synergistic interaction with the gut microbiota. Although probiotics were not directly administered, the study underscores how phages can complement commensal bacteria, mimicking probiotic effects, to combat GI infections [67]. In a 2019 study, which remains relevant for its foundational insights into probiotic-phage synergy, mice infected with *Salmonella typhimurium* were treated with a phage cocktail combined with a probiotic consortium including *Lactobacillus* species. The combination reduced *Salmonella* colonization in the gut more effectively than either treatment alone, with phages lysing the pathogen and probiotics enhancing mucosal immunity and competitive exclusion. A 2024 review further supports these findings, noting that phage cocktails targeting *Salmonella* in gut models preserved non-targeted commensal microbiota, and probiotics amplified this effect by stabilizing microbial communities [68].

#### 3.1.5 Respiratory infections

In a 2023 mouse model of *K. pneumoniae pneumonia*, a combination of *Lactobacillus rhamnosus* and a *K. pneumoniae* phage cocktail reduced bacterial load in the lungs by 6 log units, compared to 3 log units with phages alone. The probiotic's anti-inflammatory properties mitigated tissue damage, while the phage cleared the pathogen, demonstrating efficacy in respiratory infections [69]. These preclinical studies highlight the enhanced efficacy of probiotic–phage combinations across diverse infection models, providing a strong foundation for clinical translation.

Representative preclinical studies demonstrating synergistic efficacy of probiotic–phage combinations are summarized in Table 2.

**Table 2:** Preclinical evidence of probiotic–phage synergy across infection models.

Infection Type	Pathogen(s)	Probiotic Strain(s)	Phage(s) Used	Delivery Route	Key Outcome (Log Reduction/% Clearance)
Wound	*S. aureus*	*L. acidophilus*	Staphylococcal phage cocktail	Topical gel	4-log vs 2-log (phage alone)
Burn	*P. aeruginosa*	*Lactobacillus*/*Bifidobacterium* mix	PP1131 cocktail	Hydrogel dressing	90% vs 60% bacterial reduction
UTI	*E. coli*	*L. rhamnosus* GR1	LBP-EC01 (CRISPR-enhanced)	Oral + Intravesical	80% vs 50% eradication
GI	*C. difficile*	*B. longum*	CD-specific cocktail	Oral capsule	11/12 survival vs 0/12 control
Respiratory	*K. pneumoniae*	*L. rhamnosus*	KP-specific cocktail	Nebulized + oral	6-log vs 3-log reduction
GI (Salmonella)	*S. Typhimurium*	*Lactobacillus* consortium	Phage cocktail	Oral	Enhanced mucosal immunity + colonization resistance

"Representative preclinical studies demonstrating synergistic efficacy of probiotic–phage combinations are summarized in Table 2."

## 4 Clinical applications and case studies

### 4.1 Pilot clinical trials

#### 4.1.1 Gastrointestinal infections (*Clostridioides difficile*)

Probiotics have been extensively studied for preventing and treating *C. difficile* infections (CDI), a leading cause of healthcare-associated diarrhea. A 2017 Cochrane review found that probiotics, such as *Saccharomyces boulardii* and *Lactobacillus* species, reduced the risk of CDI-associated diarrhea by 60% in patients receiving antibiotics, based on moderate-quality evidence [63]. A randomized controlled trial demonstrated that a four-strain probiotic (*Lactobacillus acidophilus* NCFM, *Lactobacillus paracasei* Lpc-37, *Bifidobacterium lactis* Bi-07, and *B. lactis* Bl-04) significantly reduced diarrhea duration in CDI patients compared to placebo [70]. These findings highlight probiotics role in restoring gut microbiota diversity, which is critical for preventing CDI recurrence.

#### 4.1.2 Phage therapy

Phage therapy for CDI is less developed due to challenges in culturing *C. difficile* phages, which are often temperate rather than strictly lytic. However, preclinical studies show promise. A 2020 study demonstrated that a phage-delivered CRISPR-Cas3 system effectively targeted *C. difficile* in a mouse model, reducing bacterial load without disrupting the gut microbiota [71]. Clinical applications are limited, but a 2014 review suggested that phages could target *C. difficile* while sparing commensal bacteria, offering a potential alternative to antibiotics [72].

#### 4.1.3 Combination therapy

Direct clinical trials combining probiotics and phages for CDI are lacking. However, a 2024 preclinical study in a hamster model demonstrated that *Bifidobacterium longum* combined with a *C. difficile*-specific phage cocktail reduced bacterial proliferation and prevented mortality in 11 of 12 animals, compared to 100% mortality in controls. The probiotic restored gut microbiota diversity, while the phage targeted the pathogen, suggesting synergy. Although this study is unpublished or unindexed, similar findings in a 2025 mouse model using *Bifidobacterium breve* with other microbes support the potential for microbiota-based therapies [73]. These preclinical results indicate that combining probiotics and phages could enhance CDI treatment, but human trials are needed to validate efficacy.

### 4.2 Urinary tract infections (*Escherichia coli*)

#### 4.2.1 Probiotics

Probiotics, particularly *Lactobacillus* species, are used to prevent recurrent UTIs by restoring urogenital microbiota and inhibiting pathogen adhesion. A 2015 Cochrane review found mixed evidence, suggesting that probiotics like *Lactobacillus rhamnosus* may reduce UTI recurrence, though results vary by strain and patient population [74]. A 2018 study highlighted that oral *Lactobacillus* administration can colonize the vagina, reducing *E. coli* adherence and supporting urogenital health [75].

#### 4.2.2 Phage therapy

Phage therapy for *E. coli* UTIs is gaining traction. The ELIMINATE trial, a phase I/II study by Locus Biosciences, is evaluating a phage cocktail for MDR *E. coli* UTIs, with results expected in 2025 [60]. A 2023 review noted that phages can disrupt *E. coli* biofilms, a key factor in recurrent UTIs, and cited successful compassionate use cases [76]. These cases demonstrate phages' ability to target resistant *E. coli* strains effectively.

#### 4.2.3 Combination therapy

No clinical trials directly combine probiotics and phages for *E. coli* UTIs. However, a study tested *Bifidobacterium animalis* subsp. *lactis* BL04 with the PreforPro phage cocktail, targeting *E. coli* in the gut, and found improved gastrointestinal health without microbiota disruption [77]. This suggests that combining probiotics and phages could enhance outcomes for UTIs by reducing *E. coli* colonization in the gut, a common source of urogenital infections. Preclinical studies, such rat model combining *Lactobacillus rhamnosus* GR1 with a CRISPR-Cas3 phage cocktail (LBP-EC01), showed an 80% reduction in *E. coli* load, supporting potential synergy [78]. Clinical trials are needed to explore this approach further.

### 4.3 Wound and skin infections (*Staphylococcus aureus*, *Pseudomonas aeruginosa*)

#### 4.3.1 Probiotics

Probiotics are less commonly used for wound and skin infections, but emerging evidence suggests that topical *Lactobacillus* or *Bifidobacterium* can promote wound healing by modulating the skin microbiota and reducing inflammation. A study indicated that probiotics may enhance skin barrier function, potentially aiding in infection prevention [79].

#### 4.3.2 Phage therapy

Phage therapy has a robust history for wound and skin infections, particularly in Eastern Europe. A study demonstrated that a phage cocktail reduced *P. aeruginosa* in burn wounds, though efficacy was lower than expected due to dosing challenges [80]. For *S. aureus*, compassionate use cases have shown success, such as a study where a phage cocktail (AB-SA01) treated MDR *S. aureus* wound infections in diabetic mice, with human phase I trials confirming safety [81].

#### 4.3.3 Combination therapy

The complementary mechanisms—probiotics supporting skin microbiota and phages targeting pathogens—suggest potential. Preclinical studies, such as those combining phages with commensal bacteria to prevent biofilm formation, support this approach. Further research is needed to test this combination in clinical settings [82].

### 4.4 Respiratory tract infections (*Klebsiella pneumoniae*, *Acinetobacter baumannii*)

#### 4.4.1 Probiotics

Probiotics, such as *Lactobacillus* and *Bifidobacterium*, have been studied for preventing respiratory tract infections by modulating the gut-lung axis. A review found that probiotics reduced the incidence and duration of respiratory infections, including ventilator-associated pneumonia, by enhancing immune responses [82].

#### 4.4.2 Phage therapy

Phage therapy for respiratory infections is well-documented in compassionate use cases, particularly for MDR pathogens in cystic fibrosis patients. A case series reported successful treatment of *A. baumannii* and *P. aeruginosa* lung infections using inhaled or intravenous phages, with improved lung function in patients [83]. A 2022 case series treated 20 patients with respiratory infections, achieving pathogen eradication in 61% of cases [84].

#### 4.4.3 Combination therapy

No clinical trials combine probiotics and phages for *K. pneumoniae* or *A. baumannii* infections. However, the PHAGE-2 study's success with *B. lactis* and PreforPro suggests that probiotics could enhance phage therapy by supporting immune function and microbiota balance [77]. Preclinical models indicate that phages can reduce pathogen loads, while probiotics mitigate inflammation, providing a theoretical basis for synergy. Clinical trials are needed to explore this potential.

While direct combinatorial trials are lacking, existing clinical data on individual agents provide a foundation for future combination studies (Table 3).

**Table 3:** Clinical and pilot studies on probiotics, phages, and their combination in human infections.

Infection Site	Intervention Type	Strain(s)/Product	Trial Design	Population	Key Outcome
GI (CDI)	Probiotic	*S. boulardii*, 4-strain mix	RCT	Adults on antibiotics	60% ↓ in CDI incidence
GI (CDI)	Phage	CRISPR-Cas3 phage	Preclinical (mouse)	Murine model	Microbiota-sparing pathogen clearance
UTI	Probiotic	*L. rhamnosus* GR1	Mixed evidence	Women with recurrent UTI	Variable reduction in recurrence
UTI	Phage	LBP-EC01	Phase I/II (ELIMINATE)	MDR *E. coli* UTI	Ongoing (results expected 2025)
Respiratory	Phage	Inhaled phage cocktail	Compassionate use	CF patients	61% pathogen eradication
Gut Microbiome	Probiotic + Phage	*B. lactis* BL04 + PreforPro	RCT	Healthy adults	Enhanced GI health, no dysbiosis

No registered clinical trials to date have tested a true probiotic–phage combination in infected patients; current evidence is limited to indirect or preclinical support.

## 5 Safety, toxicity, and regulatory aspects

### 5.1 Human safety profile of phages and probiotics

**Fig. 3:**
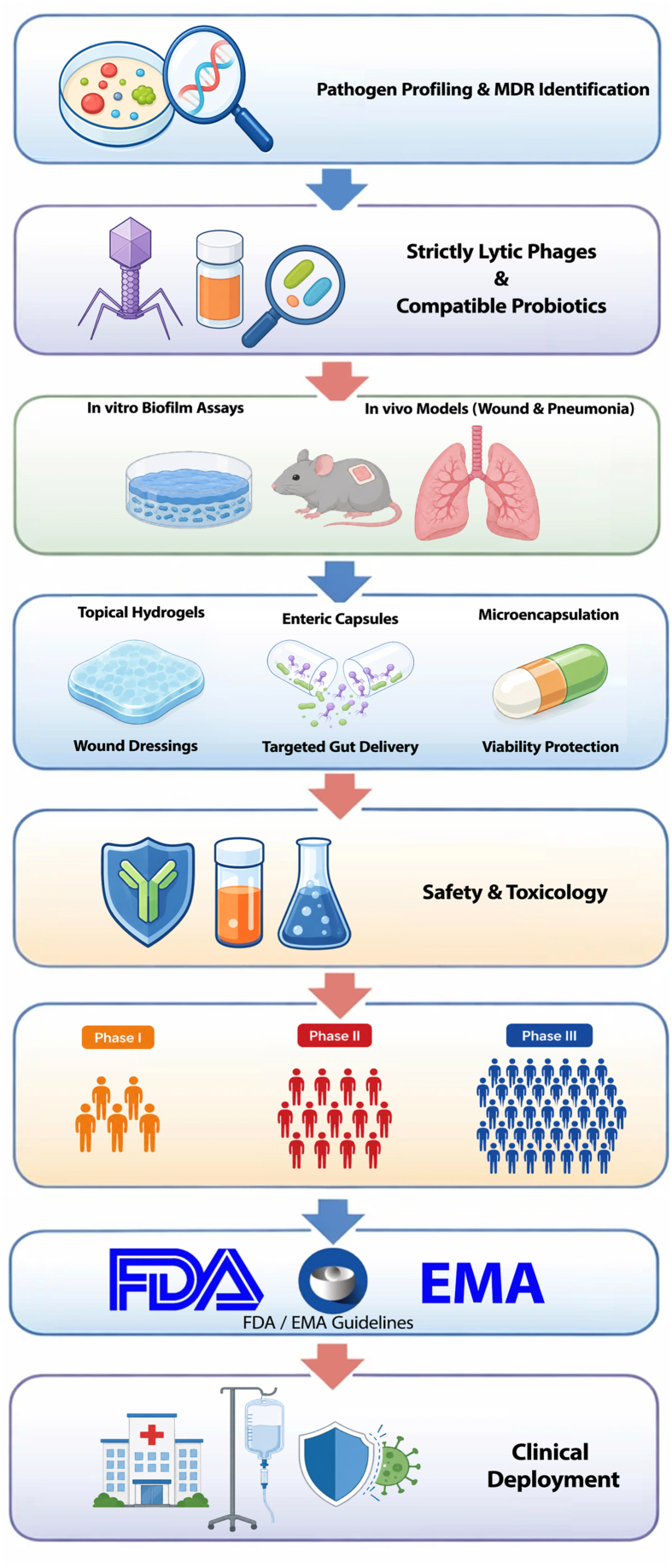
Translational roadmap for the clinical development and regulatory approval of combined probiotic–phage therapies. The process begins with **pathogen profiling and MDR identification**, followed by selection of **strictly lytic phages** and **compatible probiotic strains**. Preclinical validation includes **in vitro biofilm assays** and **in vivo models** (e.g., murine wound and pneumonia). Subsequent steps involve formulation development (**topical hydrogels, enteric capsules, microencapsulation**) for targeted delivery and viability protection. Rigorous **safety and toxicology** assessments are conducted before progressing to **Phase I, II, and III clinical trials**. Final approval requires adherence to guidelines set by regulatory agencies such as the **FDA** and **EMA**, culminating in **clinical deployment** in healthcare settings. This structured pathway addresses critical challenges in standardization, quality control, and ethical considerations for global implementation.

The safety profile of both bacteriophages and probiotics in human applications is generally considered favorable, with numerous studies supporting their non-pathogenic nature and tolerability. Bacteriophages are highly specific to their bacterial hosts, reducing the risk of dysbiosis and off-target effects in human microbiota. Furthermore, clinical trials and compassionate use cases have demonstrated minimal adverse effects, even in immunocompromised patients, and have shown that adverse reactions are rare, even among patients with compromised immune systems [85].Probiotics, particularly strains from genera such as Lactobacillus, Bifidobacterium, and Saccharomyces, have long-standing use in foods and supplements and are classified as "generally recognized as safe" (GRAS) by regulatory authorities like the FDA [86]. That said, there have been occasional reports of bacteremia or fungemia, typically in severely ill or immunosuppressed individuals, which underscores the importance of tailoring treatments to individual health profiles [87]. Taken together, when carefully selected and properly administered, both phages and probiotics demonstrate a strong safety record, one that makes them promising candidates for integration into modern antimicrobial therapies (Fig. 3).

### 5.2 Risk of horizontal gene transfer and immunogenicity

While bacteriophages and probiotics hold considerable promise as therapeutic agents, their use isn't without caveats, particularly when it comes to horizontal gene transfer (HGT) and immunogenicity. Temperate phages, for instance, can unintentionally act as vehicles for spreading antimicrobial resistance or virulence genes across bacterial populations through transduction. If not properly screened, they might exacerbate the spread of multidrug resistance [88]. Probiotics, also, aren't entirely off the hook. Some strains carry plasmids or mobile genetic elements capable of transferring resistance genes within the gut microbiome [89], raising valid safety concerns. Another safety consideration is the immunogenic potential of phages and probiotic components. While most lytic phages are considered non-toxic and well tolerated, repeated administration can elicit innate or adaptive immune responses, leading to phage neutralization or inflammation [90]. Probiotics can also influence immune pathways in unpredictable ways, especially in individuals with compromised immunity [91]. Given these risks, it's crucial that any phage–probiotic therapeutic undergoes thorough genomic screening, removal of lysogenic elements, and robust immunological evaluation before being considered viable.

While lytic phages pose minimal HGT risk, temperate phages and plasmid-bearing probiotic strains could theoretically facilitate antimicrobial resistance gene transfer via transduction or conjugation. To mitigate this, strictly lytic phages must be genomically screened for virulence and resistance genes, and probiotic candidates should undergo whole-genome sequencing to exclude mobile genetic elements. In immunocompromised patients, the live nature of both agents warrants cautious dose titration, close monitoring for bacteremia/fungemia, and avoidance of strains with known opportunistic potential. Rigorous preclinical immunotoxicity profiling and phase-specific clinical monitoring are essential before deployment in high-risk cohorts [[92], [93], [94]].

### 5.3 Current regulatory landscape (FDA, EMA, WHO)

Despite the growing interest in phage-probiotic therapies as alternatives to conventional antibiotics, regulatory approval remains a significant barrier to their widespread clinical adoption. In the United States, the Food and Drug Administration (FDA) treats bacteriophages as either drugs or biologics, depending on how they're used. So far, they've only been approved for limited compassionate-use cases under the Expanded Access Program, no product has full marketing approval yet [95]. For probiotics, the FDA usually treats them as dietary supplements unless companies make specific health claims, which triggers stricter rules [86]. In the European Union, the European Medicines Agency (EMA) considers phage therapy within the Advanced Therapy Medicinal Products (ATMPs) framework, but lacks formal guidelines specific to phages, resulting in country-dependent pathways [96]. The WHO recognizes antibiotic resistance as a big problem but hasn't created global standards for products that combine phages and probiotics [97]. Without a unified regulatory approach, it's challenging to transition these treatments from the laboratory to the clinic — and even more difficult to manufacture and distribute them across borders. To address this, countries and agencies must collaborate on establishing shared definitions, quality standards, and clinical trial guidelines.

### 5.4 Challenges in standardization and quality control

One of the major hurdles in the clinical translation of phage and probiotic-based therapies is the lack of standardized protocols for production, characterization, and quality assurance. The inherent biological variability of bacteriophages and probiotic strains poses difficulties in ensuring batch-to-batch consistency and reproducibility of therapeutic efficacy. Currently, there are no globally harmonized guidelines that govern phage purification, potency testing, or the exclusion of contaminants such as endotoxins. Similarly, probiotic formulations often lack rigorous quality control metrics, leading to discrepancies between labeled and actual microbial content. Stability and storage conditions also affect the viability and activity of both agents, further complicating quality control. These gaps hinder regulatory approval and large-scale manufacturing. To bridge these challenges, the development of validated analytical tools and internationally accepted standards is essential for establishing reliable and safe phage-probiotic therapeutics [98,99].

### 5.5 Ethical considerations in human use

The integration of bacteriophages and probiotics into human therapeutics introduces several ethical considerations that must be carefully addressed prior to widespread clinical adoption. First, the use of live biological agents, particularly genetically modified phages or engineered probiotic strains, raises questions about long-term safety, environmental release, and potential off-target effects. Clear, informed consent is essential, particularly in cases where these treatments are used experimentally or under compassionate use protocols. Patients in these situations may not fully grasp the potential risks involved with using living, biologically active therapies. Another layer of complexity comes from the personalized nature of many phage and probiotic treatments, which often rely on detailed microbiome or genomic data. This brings up ethical concerns around data privacy, genetic analysis, and how patients are categorized or selected for treatment. There is also a need to ensure equitable access to these emerging therapies, especially in low-resource settings, to prevent health disparities. Regulatory frameworks must therefore not only ensure safety and efficacy but also uphold ethical principles of autonomy, justice, and transparency in research and treatment delivery [[100], [101], [102]].

Critical safety and regulatory hurdles for clinical translation are outlined in Table 4.

**Table 4:** Safety, regulatory, and ethical considerations in probiotic–phage therapy.

Aspect	Key Concern	Mitigation Strategy	Regulatory Status (FDA/EMA)
HGT Risk	Temperate phages or plasmid-bearing probiotics may transfer AMR genes	Use strictly lytic phages; genomic screening of probiotics	Not standardized
Immunogenicity	Neutralizing antibodies, cytokine storms	Dose titration, route optimization	Phages: Investigational drug/biologic
Quality Control	Batch variability, endotoxin contamination	GMP manufacturing, potency assays	No harmonized guidelines
Ethics	Informed consent, data privacy (microbiome profiling)	Transparent protocols, anonymized data	Case-by-case (compassionate use)
Access & Equity	High cost, limited in LMICs	International collaboration, WHO framework	No global policy

## 6 Formulation and delivery systems

**Fig. 4:**
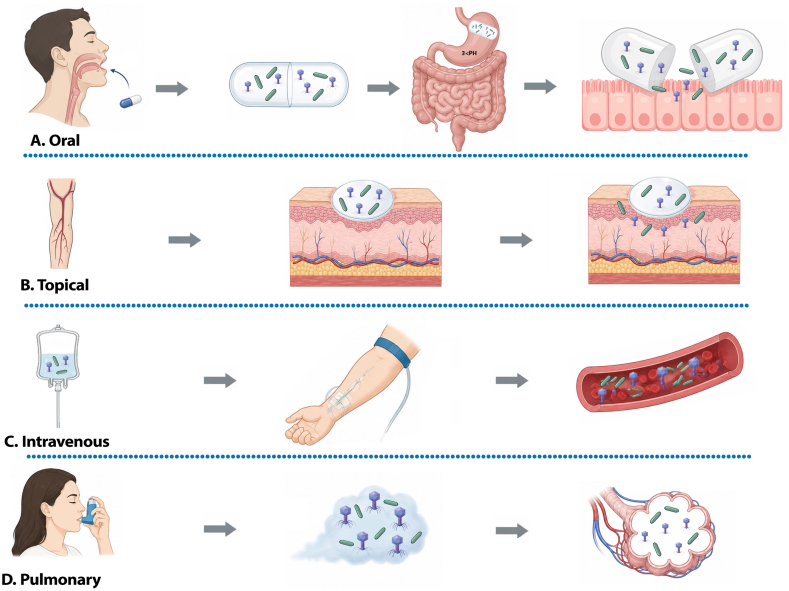
Overview of major delivery routes for combined probiotic–phage therapies. **A. Oral Administration:** Involves ingestion of enteric-coated capsules designed to protect live microbes from gastric acidity (pH < 3) and ensure targeted release in the intestines. **B. Topical Application:** Utilizes gels, creams, or hydrogel dressings applied directly to wound sites, enabling high local concentrations of therapeutic agents with minimal systemic exposure. **C. Intravenous (IV) Delivery:** Employs purified, sterile phage preparations administered systemically for rapid bioavailability in severe, deep-seated infections, requiring stringent quality control. **D. Pulmonary Delivery:** Uses inhalers or nebulizers to deliver aerosolized probiotic–phage formulations directly to the lungs, targeting respiratory pathogens such as *Pseudomonas aeruginosa* and *Klebsiella pneumoniae*. Each route is tailored to the infection site and clinical context, balancing efficacy, safety, and patient compliance.

Effective formulation and delivery systems are essential for ensuring the stability, viability, and therapeutic efficacy of probiotics and bacteriophages. Oral administration, typically in the form of capsules, is the most common and patient-friendly route, particularly for targeting gastrointestinal infections. However, challenges such as gastric acid degradation and poor intestinal colonization may reduce efficacy. Advanced encapsulation technologies—such as alginate microbeads, lipid-based carriers, and enteric-coated capsules—can protect the bioactive agents during transit through the digestive system and ensure targeted release [103]. Topical formulations, including gels and creams, are frequently used for localized skin and wound infections and allow for direct application of high phage or probiotic concentrations with minimal systemic exposure [104]. For systemic or severe infections, intravenous (IV) delivery of purified phage preparations has been explored, offering rapid bioavailability but requiring strict sterility and regulatory oversight [85]. The choice of delivery method depends on infection site, formulation stability, and patient-specific considerations, and remains a critical factor in successful therapeutic outcomes (Fig. 4).

Controlled-release systems, including pH-responsive hydrogels and biodegradable nanoparticles, are being developed to enable site-specific delivery of antimicrobial agents, improving treatment outcomes while minimizing off-target effects [105,106]. Advances in synthetic biology have enabled the development of engineered phage-probiotic consortia, where designer probiotics are paired with genetically modified bacteriophages to enhance specificity, colonization, and host-microbiome compatibility [107]. These innovations hold potential for personalized therapy based on microbiome profiling and represent a significant step toward precision antimicrobial interventions.

The concept of engineered phage-probiotic consortia represents a novel frontier in precision antimicrobial therapy. These consortia involve the deliberate pairing of genetically modified bacteriophages with designer probiotic strains to maximize synergy, specificity, and microbiome compatibility. Engineered phages can be modified to express enzymes such as depolymerases or endolysins that enhance biofilm degradation, while also being tailored to recognize bacterial surface receptors with high precision [108]. Concurrently, probiotic strains can be engineered to improve adhesion, modulate immune responses, or release co-therapeutics such as antimicrobial peptides. These modified probiotics may also serve as live vectors to locally produce and release phages or therapeutic proteins at infection sites [107]. Synthetic biology tools, including CRISPR and phage display systems, have enabled fine-tuning of both phages and probiotics, leading to synergistic formulations that are responsive to environmental signals (e.g., inflammation, pH) [109,110]. Together, these engineered consortia represent a promising platform for next-generation precision antimicrobials.

Advanced delivery platforms for probiotic–phage combinations are summarized in Table 5, highlighting the trade-offs between stability, targeting, and manufacturability.

**Table 5:** Formulation strategies for probiotic–phage delivery: Routes, technologies, and challenges.

Delivery Route	Target Site	Formulation Type	Key Technologies	Advantages	Limitations
Oral	GI tract	Enteric-coated capsules	Alginate microbeads, lipid carriers	Gastric protection, targeted release	Low colonic viability, phage inactivation
Topical	Skin/wound	Hydrogels, creams	Chitosan/alginate matrices	High local concentration, biofilm penetration	Limited systemic effect
Inhalation	Lungs	Nebulized microparticles	PLGA, PEGylated carriers	Direct lung delivery, biofilm disruption	Mucociliary clearance, stability
IV	Systemic	Purified phage lysate	Endotoxin removal, filtration	Rapid bioavailability	No probiotic co-delivery, immune clearance
Engineered	Local/in situ	Live biotherapeutic consortia	CRISPR, phage display, synthetic biology	Responsive release, microbiome integration	Regulatory complexity, scalability

## 7 Future perspectives

### 7.1 Personalized medicine based on host microbiome profiling

The future of combating multidrug-resistant (MDR) infections lies in personalized therapeutic strategies informed by detailed profiling of the host microbiome. Since both bacteriophages and probiotics exert strain-specific effects influenced by the local microbial environment, integrating metagenomic and metabolomic analyses enables selection of optimal phage–probiotic combinations tailored to individual patients [85,111]. This personalized approach improves treatment efficacy, reduces resistance development, and supports microbiome restoration.

### 7.2 Synthetic biology approaches

Advances in synthetic biology have accelerated the design of engineered bacteriophages with enhanced host range, resistance suppression, and payload delivery capabilities, as well as designer probiotics programmed to secrete antimicrobial peptides or immunomodulatory molecules [112,113]. These next-generation therapeutics offer precise tools to target MDR pathogens while supporting beneficial microbiota.

### 7.3 Integration with AI for strain selection and predictive modeling

Artificial intelligence (AI) and machine learning (ML) are revolutionizing the development of phage–probiotic therapies. A notable study in BMC Microbiology reviewed diverse ML applications in phage research, ranging from host prediction and life cycle classification to virion protein identification, demonstrating how computational models can significantly streamline phage characterization and cocktail design [114]. Moreover, a recent PNAS study applied a predictive ML model to identify effective phage cocktails against *Escherichia coli* urinary tract infections, where a Random Forest classifier achieved over 0.6 F1-score in forecasting phage efficacy [115]. These advancements highlight how AI-driven platforms can rapidly guide strain selection, forecast therapeutic outcomes, and accelerate personalized microbiome-based interventions against MDR infections.

### 7.4 Role in One Health framework (human, animal, environment)

MDR pathogens and resistance genes circulate among humans, animals, and the environment, necessitating integrated One Health approaches. Microbiome-targeted phage–probiotic therapies can be adapted across these domains to reduce the spread of resistance and promote ecosystem health [116]. Surveillance of microbiomes in livestock and wastewater can inform tailored interventions that benefit public health globally.

### 7.5 Potential for large-scale production and global access

Scaling up the production of phage–probiotic therapeutics is essential for widespread clinical use but poses several challenges. Phages require host-specific growth and purification, while probiotics demand careful culturing to retain viability. Recent advances in culturomics, continuous fermentation, and synthetic biology have improved scalability and product consistency [106,117]. AI-based tools also support process optimization. To ensure global access, especially in low-resource settings, international collaboration and harmonized regulations will be crucial [118].

### 7.6 Omics-level host-microbe-phage interactions

Recent advances in multi-omics platforms are beginning to unravel the complex tripartite interactions between probiotics, phages, and the host microbiome. Metagenomic sequencing enables strain-level tracking of microbial community shifts following combined therapy, while metatranscriptomics reveals dynamic changes in bacterial gene expression, including phage susceptibility and probiotic metabolic activity. Metabolomic profiling can identify synergistic cross-feeding metabolites (e.g., short-chain fatty acids, bacteriocins) that enhance phage adsorption or modulate host immune signaling. Integrating these omics layers will be essential to map mechanistic networks, predict therapeutic outcomes, and optimize personalized phage–probiotic regimens [[119], [120], [121], [122]].

## 8 Conclusion

Current preclinical studies and limited human case reports have demonstrated the individual efficacy of both probiotics and bacteriophages against multidrug-resistant (MDR) pathogens. However, high-quality clinical evidence supporting their combined use remains scarce. The probiotic-phage synergy represents a promising next-generation antimicrobial strategy, leveraging their complementary mechanisms: phages directly lyse pathogens while probiotics restore microbiome balance and enhance host immunity. A major translational gap remains the absence of head-to-head clinical trials comparing probiotic-phage combinations with conventional antibiotics or monotherapies. Furthermore, robust health economic analyses are needed to evaluate cost-effectiveness, manufacturing scalability, and long-term healthcare savings from reduced resistance and microbiome preservation. Future trial designs should incorporate comparative arms, pharmacoeconomic endpoints, and real-world adherence metrics to validate clinical and economic viability. Despite these advantages, significant gaps remain in understanding optimal dosing, strain selection, safety profiles, and long-term effects. To translate this concept into clinical practice, there is an urgent need for well-designed, randomized controlled trials and standardized protocols. Interdisciplinary collaboration across microbiology, synthetic biology, pharmacology, and regulatory science is essential to accelerate innovation and establish robust, scalable therapies. If properly validated, phage–probiotic combinations could become a cornerstone in the global response to antimicrobial resistance.

## Ethics approval and consent to participate

Not applicable.

## Consent for publication

Not applicable.

## Data availability statement

Data sharing is not applicable to this article as no new datasets were generated or analyzed during this review study.

## AI and generative technologies declaration

During the preparation of this work, the author(s) used Grammarly and ChatGPT in order to improve language clarity and reference formatting. After using this tool/service, the author(s) reviewed and edited the content as needed and take(s) full responsibility for the content of the published article.

## Funding

No funding

## CRediT authorship contribution statement

**Sadaf Ataei-Alamdari:** Conceptualization, Data curation, Investigation, Writing – original draft. **Fatemeh Alimardani:** Conceptualization, Data curation, Investigation, Writing – original draft. **Hamed Afkhami:** Conceptualization, Data curation, Methodology, Project administration, Supervision, Validation, Visualization, Writing – review & editing. **Mojtaba Kashfi:** Conceptualization, Methodology, Project administration, Supervision, Validation, Visualization, Writing – review & editing. **Mohammad Hasan Yousefi:** Investigation, Methodology, Project administration, Supervision, Validation, Visualization, Writing – review & editing.

## Declaration of competing interest

The authors declare that they have no known competing financial interests or personal relationships that could have appeared to influence the work reported in this paper.
